# Multiomics Analysis of Endocytosis upon HBV Infection and Identification of SCAMP1 as a Novel Host Restriction Factor against HBV Replication

**DOI:** 10.3390/ijms23042211

**Published:** 2022-02-17

**Authors:** Tanzeel Yousaf, Yuting Sun, Wajeeha Naz, Yang Liu, Jiaqi Xu, Sen Yuan, Kangwei Wu, Min Wang, Jun Wang, Mingxiong Guo, Guihong Sun

**Affiliations:** 1 School of Basic Medical Sciences, Wuhan University, Wuhan 430071, China; tanzyousaf@gmail.com (T.Y.); wajeehanaz99@gmail.com (W.N.); ly326481949@163.com (Y.L.); 2018203010058@whu.edu.cn (J.X.); yuansen61@163.com (S.Y.); 2019203010008@whu.edu.cn (K.W.); min.wang@whu.edu.cn (M.W.); 2 Hubei Key Laboratory of Cell Homeostasis, College of Life Sciences, Wuhan University, Wuhan 430072, China; syt_282805747@163.com (Y.S.); guomx@whu.edu.cn (M.G.); 3 National “111” Center for Cellular Regulation and Molecular Pharmaceutics, Hubei University of Technology, Wuhan 430068, China; jun_wang@hbut.edu.cn; 4 Hubei Provincial Key Laboratory of Allergy and Immunology, Wuhan University, Wuhan 430071, China

**Keywords:** HBV, SCAMP1, transcriptome, proteome, ubiquitylome, inhibition, replication

## Abstract

Hepatitis B virus (HBV) infection remains a major global health problem and the primary cause of cirrhosis and hepatocellular carcinoma (HCC). HBV intrusion into host cells is prompted by virus–receptor interactions in clathrin-mediated endocytosis. Here, we report a comprehensive view of the cellular endocytosis-associated transcriptome, proteome and ubiquitylome upon HBV infection. In this study, we quantified 273 genes in the transcriptome and 190 endocytosis-associated proteins in the proteome by performing multi-omics analysis. We further identified 221 Lys sites in 77 endocytosis-associated ubiquitinated proteins. A weak negative correlation was observed among endocytosis-associated transcriptome, proteome and ubiquitylome. We found 33 common differentially expressed genes (DEGs), differentially expressed proteins (DEPs), and Kub-sites. Notably, we reported the HBV-induced ubiquitination change of secretory carrier membrane protein (SCAMP1) for the first time, differentially expressed across all three omics data sets. Overexpression of SCAMP1 efficiently inhibited HBV RNAs/pgRNA and secreted viral proteins, whereas knockdown of SCAMP1 significantly increased viral production. Mechanistically, the EnhI/XP, SP1, and SP2 promoters were inhibited by SCAMP1, which accounts for HBV X and S mRNA inhibition. Overall, our study unveils the previously unknown role of SCAMP1 in viral replication and HBV pathogenesis and provides cumulative and novel information for a better understanding of endocytosis in response to HBV infection.

## 1. Introduction

Hepatitis B virus (HBV) is a major threat to human health, and it is estimated that there are 350–500 million people affected with chronic HBV infection worldwide. [1,2]. Viruses exploit the abilities of the host cell by manipulating different cellular pathways; they can activate and modify these pathways for their specific purposes and spread. Viruses have evolved strategies to integrate several host factors to penetrate and escape the endosomal membrane [3,4]. HBV internalization into cells is triggered by virus–receptor interactions in clathrin-mediated endocytosis (CME). Indeed, the preS1 domain of HBV envelope proteins has been shown to interact with clathrin and protein adaptor 2 (AP-2) during entering into immortalized human primary hepatocytes [5,6]. Recently, it has been shown that the endocytosis-associated protein epidermal growth factor receptor (EGFR) mediates the internalization of sodium taurocholate co-transporting polypeptide (NTCP)-bound HBV via its endocytosis/sorting pathway [7]. However, many aspects and the significance of how infection alters the host endocytosis process remained completely obscure [8,9].

Secretory Carrier Membrane Proteins (SCAMPs) are a family of ubiquitous tetraspanning, integral membrane proteins that play a vital role in mediating exocytosis and membrane trafficking systems [10,11]. There are five genes of SCAMPs discovered in mammals [12]. SCAMP1 is the most abundant protein among all the discovered SCAMP’s and appears to be present at high levels on synaptic vesicles [13]. SCAMP1 is highly up-regulated in cervical, pancreatic, and gallbladder cancer tissues [14]. SCAMP3 expression dramatically increased in HCC tissues, and its knockdown led to the suppression of cell proliferation of HCC cells [15]. Recent studies reported that SCAMP2/4/5 could be potential prognostic markers for acute myeloid leukemia (AML) [16]. SCAMP5 is associated with intellectual disability (ID) and autistic spectrum disorder (ASD).

In this study, we performed RNA sequencing, quantitative mass spectrometry, and Ubiscan analysis in HepG2.2.15 and HepG2 cell lines in three distinct biological replicates that were proceeded independently to explore HBV-mediated alterations in endocytosis associated proteins. We discovered the previously unknown antiviral function of endocytosis-associated protein SCAMP1 against HBV replication. Briefly, our findings provide valuable information to develop novel therapeutics and a comprehensive understanding of alterations of endocytosis-associated proteins in response to HBV infection.

## 2. Results

### 2.1. HBV Integration Changes Endocytosis Associated Transcriptome Profile in the HepG2.2.15 Cell Line

**Figure 1:**
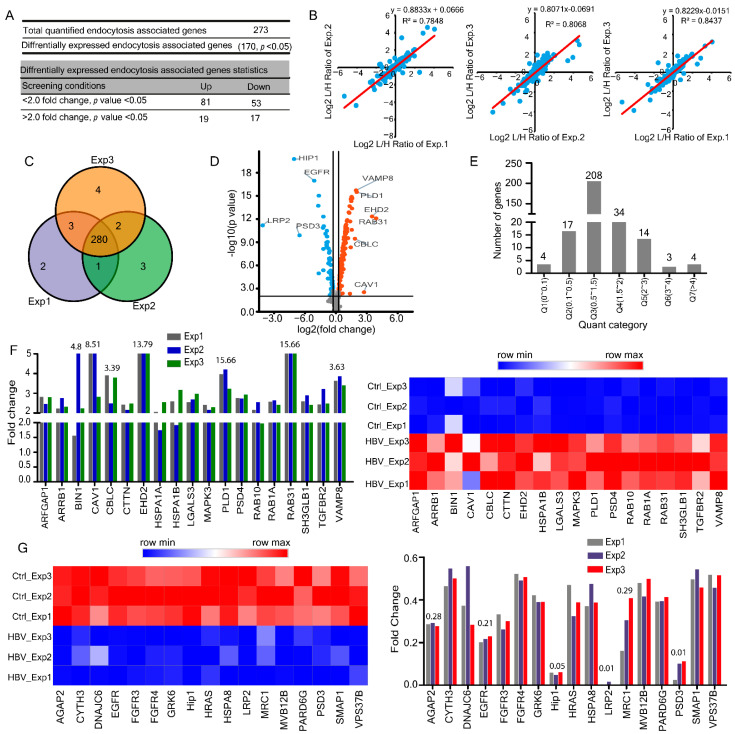
HBV integration modifies host endocytosis-associated transcriptome in HepG2.2.15 cells. (**A**) Summary of quantified and differentially expressed transcriptome. (**B**) Pearson’s correlation (R^2^) plots for three representative experiments from HepG2.2.15 cells. (**C**) Venn diagrams for three representative transcriptome experiments in HepG2.2.15, gene numbers are indicated. (**D**) The identified endocytosis-associated DEGs are shown in a volcano plot. (**E**) Distribution of regulated genes quantile results. (**F**) Two-fold up-regulated genes from three biological replicates in HepG2.2.15 cells along with heatmap representation. (**G**) Two folds down-regulated genes from three biological replicates in HepG2.2.15 cells, along with heatmap representation.

In our previous study, we found that HBV induced significant alterations in the host global ubiquitylome and proteome [17]. However, a comprehensive view of the cellular endocytosis-associated transcriptome, proteome and ubiquitylome in response to HBV infection is lacking. Therefore, we thoroughly explored the differentially regulated genes (DEGs) involved in the endocytosis pathway via functional annotation and manual data inspection from previous reports [18]. In transcriptome, we found 170 DEGs (*p* ≤ 0.05) out of 273 quantified genes, 100 DEGs were up-regulated while 70 were down-regulated, 36 genes were changed more than two folds (19 up-regulated and 17 down-regulated) (Figure 1A and Supplementary Table S2). A strong Pearson’s correlation coefficient of 0.81 was observed across the genes in three different biological replicates of HepG2.2.15 and HepG2 cell lines that proceeded independently (Figure 1B). Venn diagram demonstrated significant overlap across the biological triplicates (Figure 1C). Volcano plot indicated differentially expressed DEGs, including highly expressed VAMP8, PLD1, EHD2, RAB31, CBLC, and significantly down-regulated HIP1, EGFR, LRP2, and PSD3 (Figure 1D). Notably, the endocytic pathway associated with SCAMPs family members was differentially expressed (*p* ≤ 0.05), SCAMP2 ≈ 1.21 and SCAMP4 ≈ 1.12 were up-regulated while SCAMP1 ≈ 0.70, SCAMP3 ≈ 0.59, and SCAMP5 ≈ 0.82 were down-regulated (Supplementary Table S2).

All quantified and differentially expressed genes were segregated into seven quantiles (Q1–Q7) according to their fold change Q1 (0 < 0.1), Q2 (0.1<0.5), Q3 (0.5 < 1.5), Q4 (1.5 < 2), Q5 (2< 3), Q6 (3 < 4), Q7 (> 4) (Figure 1E). Among the two-fold up-regulated endocytosis-associated genes in response to HBV infection, the markedly expressed ones were RAB31 ≈ 15.66, EHD2 ≈ 13.79, CAV1 ≈ 8.51, BIN1 ≈ 4.82, PLD1 ≈ 3.80, VAMP8 ≈ 3.63, CBLC ≈ 3.39 folds (Figure 1F). In a similar context, distinctly down-regulated genes were LRP2 ≈ 0.01, PSD3 ≈ 0.1, HIP1 ≈ 0.055, EGFR ≈ 0.21, AGAP2 ≈ 0.28, MRC1 ≈ 0.29 across two folds down-regulated transcriptome (Figure 1G). Overall, the transcriptomic data showed remarkable consistency across 273 quantified genes, including highly expressed VAMP8, PLD1, EHD2, RAB31 and CBLC.

### 2.2. HBV Induced Alterations in Host Endocytosis Associated Proteome

**Figure 2:**
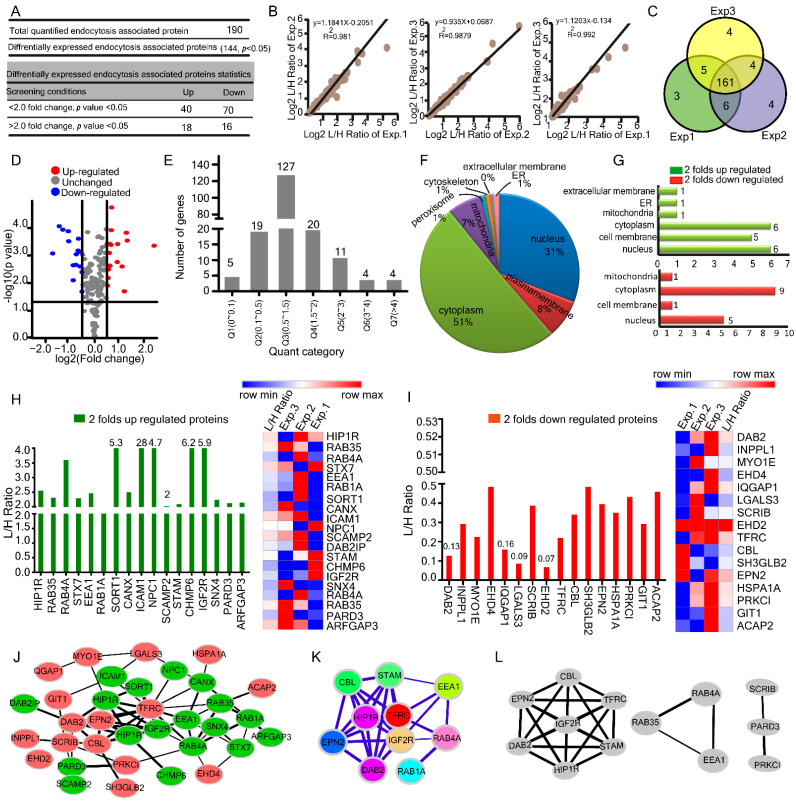
HBV infection changes endocytosis associated host proteome profile in HepG2.2.15 cells. (**A**) Summary of quantified and differentially expressed proteins and peptides. (**B**) Pearson’s correlation (R^2^) plots for three representative experiments from HepG2.2.15 cells. (**C**) Venn diagrams of peptides in HepG2.2.15 with peptide numbers indicated. (**D**) The identified endocytosis-associated DEPs are shown in a volcano plot (**E**) Distribution of regulated proteins quantification results. (**F,G**) The subcellular localization of differentially expressed and two folds up & down-regulated proteins. (**H**) L/H ratios for two folds up-regulated proteins from three biological replicates in HepG2.2.15 cells along with heatmap representation. (**I**) L/H ratios for two folds of down-regulated proteins from three biological replicates in HepG2.2.15 cells along with heatmap representation. (**J**)Protein-protein interaction network constructed with the up-and down-regulated DEPs. Green nodes represent up-regulated proteins, and red nodes represent down-regulated genes. DEPs = differently expressed proteins. (**K**) The defined hub genes were identified by the CytoHubba plugin by degree method through DEPs PPI network. (**L**) The most significant module selected by the MCODE plugin, comprising 7 nodes and 21 edges.

To better understand the effects of HBV on the endocytosis pathway, we analyzed the proteome data, and we found that 144 (*p* ≤ 0.05) proteins were differentially expressed out of 190 quantified proteins involved in the endocytosis pathway response to HBV infection. Across these proteins, 34 changed over two folds, which were 18% of total quantified proteins (18 up-regulated with light: heavy [L: H] ratios ≥ 2.0 and 16 down-regulated with light: heavy [L: H] ratios ≤ 0.5) and 110 proteins changed below two folds (40 up-regulated and 70 down-regulated) (Figure 2A and Supplementary Table S3). Pearson’s correlation coefficients of up to 0.98 indicated high reproducibility (Figure 2B) consistent with significant overlap across biological triplicates representing close imitation among biological triplicate samples (Figure 2C). The volcano plot shows the identified differentially expressed proteins (DEPs) (Figure 2D). All differentially expressed proteins were divided into seven quantiles (Q1–Q7) according to their light: heavy [L: H] ratios Q1 (0 < 0.1), Q2 (0.1< 0.5), Q3 (0.5 < 1.5), Q4 (1.5 < 2), Q5 (2< 3), Q6 (3 < 4), Q7 (> 4, the maximum number of proteins were found to be 127 in Q3 (0.5 < 1.5) (Figure 2E). Gene ontology annotation was applied to classify proteins in their subcellular localization (Figure 2F). According to the sub-cellular localization analysis, the cytoplasm contributed approximately 51% to total cellular protein mass, surpassing nucleus (31%), plasma membrane (8%), and mitochondria (7%) with relatively minor contributions from peroxisomes, ER, and cytoskeleton. Subsequently, we found that the up-regulated proteins were highly enriched in nucleus 30%, cytoplasm 30%, and cell membrane 25% (Figure 2G, top), whereas down-regulated proteins were enriched in cytoplasm 56.25% (Figure 2G, bottom), indicating that HBV promotes the down-regulation of proteins in the cytoplasm.

Gene ontology (GO) enrichment-based clustering analysis showed notably up-regulated endocytosis associated proteins which changed over two-fold in response to HBV infection were ICAM1 ≈ 28.43, CHMP6 ≈ 6.21, IGF2R ≈ 5.97, SORT1 ≈ 5.34, NPC1 ≈ 4.71, SCAMP2 ≈ 2.079 andSCAMP1 ≈ 1.41 (Figure 2H). Importantly, ICAM1 was found to be highly up-regulated in proteome with an L/H ratio of 28.43. Intercellular adhesion molecule (ICAM-1) is highly expressed in chronic hepatitis B infection and is associated with hepatocellular carcinoma [19,20]. Among the proteins that changed two folds or more in response to HBV infection, the markedly down-regulated endocytosis-associated proteins were DAB2 ≈ 0.13, IQGAP1 ≈ 0.16, LGALS3 ≈ 0.09, and EHD2 ≈ 0.07 folds (Figure 2I).

Viral infections are mediated by several protein-protein interactions (PPIs), which can be represented as networks (protein interaction networks, PINs), with proteins being depicted as nodes, and their interactions as edges [21]. So, for the inspection of interaction among the DEGs, we constructed a protein-protein interaction (PPI) network using Cytoscape, with 34 nodes and 66 edges based on endocytosis associated 34 proteins which changed over two folds in response to HBV infection (Figure 2J). CytoHubba of Cytoscape was used to identify and select hub proteins. The high degree protein was deemed the hub protein with an essential biological function. The top ten nodes with the highest degrees included transferrin receptor protein 1 (TFRC, score = 14), insulin-like growth factor II (IGF2R, score = 9), huntingtin interacting protein 1-related protein (HIP1R score = 8), disabled homolog 2 (DAB2, score = 8), E3 ubiquitin-protein ligase (CBL score = 8), signal transducing adaptor molecule (STAM, score = 8), Ras-related protein Rab-4A (RAB4A score = 8), Early endosome antigen 1 (EEA 1 score = 7), epsin 2 (EPN2 score = 6) and Ras-related protein Rab-1A (RAB1A score = 5). kAmong the identified hub proteins IGF2R, 

HIP1R, STAM, RAB4A, RAB1A and EEA1were up-regulated while TFRC, DAB2, CBL and EPN2 were down-regulated in our proteomic analysis (Figure 2K). It has been suggested that viral proteins establish interactions with more central and highly interacted host proteins [21]. In addition, the MCODE of Cytoscape detected three modules, the most significant module with a score ≥ 4, which consisted of 7 nodes and 21 edges (Figure 2L). Collectively, proteome analysis reveals 190 endocytosis-related quantified proteins, most of which are sub-cellularly localized in the cytoplasm.

### 2.3. Changes in Host Endocytosis-Associated Ubiquitylome upon HBV Infection

**Figure 3:**
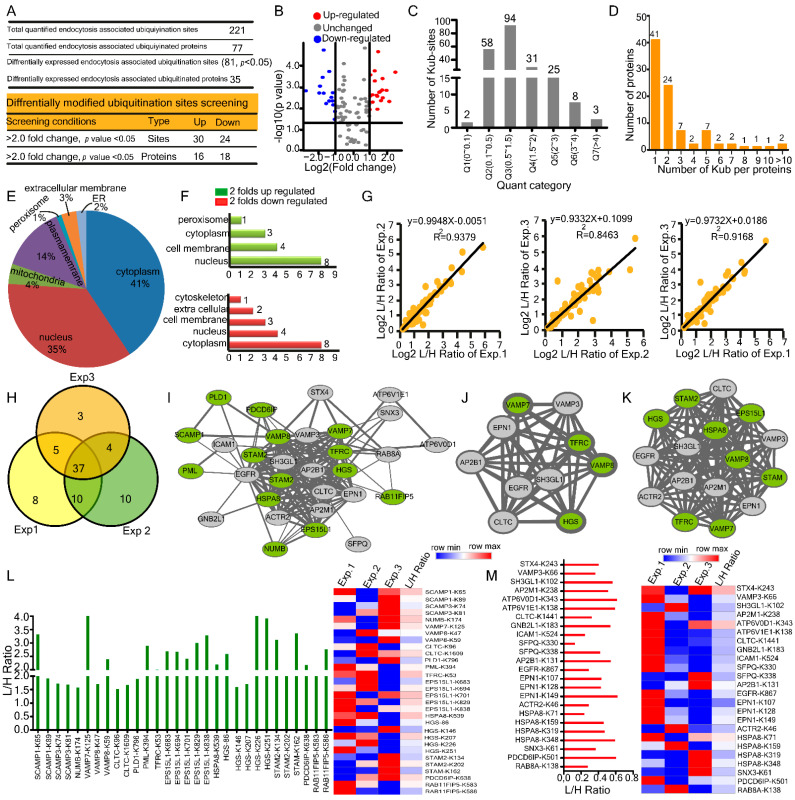
Host endocytosis associated ubiquitylome modifications upon HBV infection. (**A**) Summary of quantified and differentially expressed proteins and Kub-sites. (**B**) The identified endocytosis associated Kub-sites are shown in a volcano plot. (**C**) Distribution of regulated Kub-sites quantification results. (**D**) Association between the number of proteins and the number of Kub-sites per protein is shown. (**E,F**) The subcellular localization of differentially expressed and two folds up &down-regulated proteins. (**G**) Pearson’s correlation (R^2^) plots for three representative experiments from HepG2.2.15 cells. (**H**) Venn diagrams for three representative experiments of Kub-sites in HepG2.2.15 cells, Kub-sites numbers are indicated (**I**) Protein-protein interaction network constructed with the up and down-regulated ubiquitinated DEPs, green nodes represent up-regulated ubiquitinated proteins, and grey nodes represent down-regulated. DEPs = differently expressed proteins. (**J**) The defined hub genes identified by CytoHubba plugin by degree method through ubiquitinated DEPs PPI network. (**K**) The most significant module selected by the MCODE plugin, comprising 16 nodes and 120 edges. (**L**) L/H ratios for two folds up-regulated Kub-sites from three biological replicates in HepG2.2.15 cells along with heatmap representation. (**M**) L/H ratios for two folds down-regulated Kub-sites from three biological replicates in HepG2.2.15 cells along with heatmap representation.

In the ubiquitylome, we quantified a total of 221 ubiquitinated Lys sites in 77 endocytosis-associated proteins, whereas 81 lysine sites were differentially expressed in 35 ubiquitinated proteins. Among these ubiquitinated Lys sites, 54 were changed over two folds, including 30 up-regulated and 24 down-regulated (Figure 3A and Supplementary Table S4). The volcano plot displayed the differentially expressed lysine ubiquitination sites (Kub-sites) (Figure 3B). All quantified and differentially expressed Kub-sites were divided into seven quantitative categories Q1 (0 < 0.1), Q2 (0.1< 0.5), Q3 (0.5 < 1.5), Q4 (1.5 < 2), Q5 (2< 3), Q6 (3 < 4), Q7 (> 4), the highest number of Kub-sites was found to be 94 in category Q3 (0.5 < 1.5) (Figure 3C). There were 41 proteins whose ubiquitination was modified at a single lysine position. Twenty four proteins were found to be ubiquitinated at two lysine sites (Figure 3D). According to the sub-cellular localization analysis, the cytoplasm contributed approximately 41% to total cellular ubiquitinated protein mass, exceeded nucleus (35%), plasma membrane (14%), and mitochondria (4%) with comparatively insignificant contributions from peroxisomes, ER, and extracellular membrane (Figure 3E). Afterward, we found that the up-regulated ubiquitinated proteins were highly enriched in the nucleus 50%, cell membrane 25%, and cytoplasm 18% (Figure 3F, top). In contrast, down-regulated ubiquitinated proteins were enriched in cytoplasm 44% (Figure 3F, bottom), signifying that HBV promotes the down-regulation of proteins in the cytoplasm. Pearson’s correlation coefficients of up to 0.90 reflected high correlations across the ubiquitinated proteins (Figure 3G). Similarly, the Venn diagram shows the significant overlap of quantified proteins across biological triplicates (Figure 3H).

Both pathogen and cellular proteins constantly compete for binding partners and interactions during a viral infection. Identifying and blocking such interactions is the main mechanism underlying antiviral therapies from a biomedical perspective. Therefore, we conducted a PPI analysis of ubiquitinated proteins to assess and integrate the interactive relationships among the DEGs [21].The protein-protein interaction (PPI) network of ubiquitinated proteins comprised of 30 nodes and 161 edges (14 up-regulated and 17 downregulated proteins) (Figure 3I). SCAMP1, one of the vital endocytosis-associated proteins, was highly up-regulated with 3 edges (interacts with VAMP8, PLD1, and EGFR). Using Cytohubba of Cytoscape, we determined hub proteins with the highest degree included Epidermal growth factor receptor (EGFR, score = 22) (Figure 3J). Furthermore, MCODE analysis showed a single module with a high score ≥ 16, which consisted of 16 nodes and 120 edges (Figure 3K). Furthermore, we reported remarkably ubiquitinated endocytosis-associated proteins and their respective Kub-sites in response to HBV infection were SCAMP1-K65, VAMP7-K125, VAMP8-K59, PML-K394, EPS15L1-K683, HSPA8-K539, HGS-K226, STAM2-K134, STAM-K162, PDCD6IP-K638, and RAB11FIP5-K586 (Figure 3L). HBV mediated significantly down-regulated ubiquitinated endocytosis associated proteins, and their Kub-sites were VAMP3-K66, ATP6V0D1-K343, ATP6V1E-K138, EPN1-K149, HSPA8-K348, and PDCD6IP-K501 (Figure 3M). Altogether, in the ubiquitylome, we found 221 ubiquitinated Lys sites in 77 endocytosis-associated proteins, with 41 proteins ubiquitinated at a single lysine location.

### 2.4. Comparison and Correlation among Transcriptome, Proteome, and Ubiquitylome

**Figure 4:**
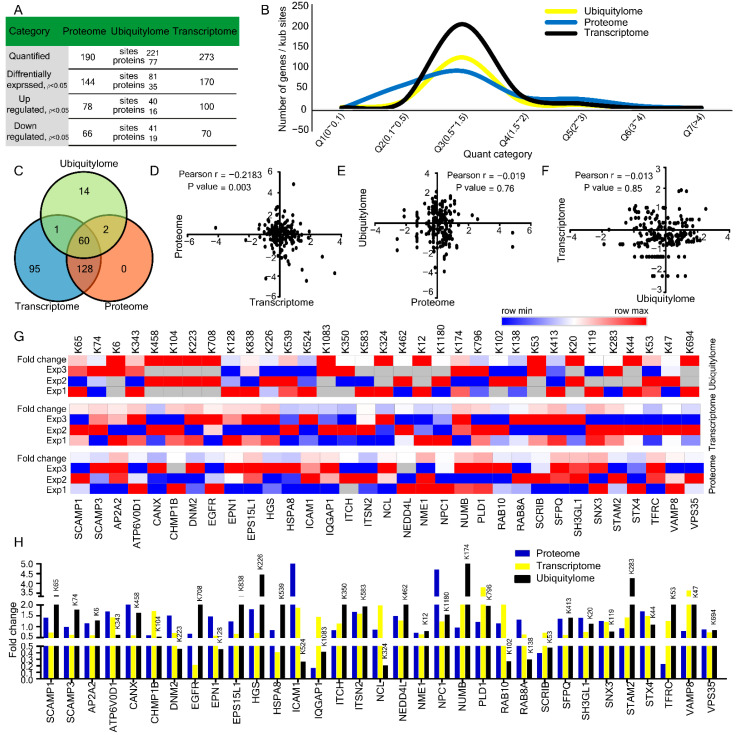
Comparison and correlation of transcriptome, proteome, and ubiquitylome changes. (**A**) Summary of quantified and differentially expressed genes, proteins, and Kub-sites. (**B**) Comparison of the distribution of quantified transcriptome, proteome, and ubiquitylome. (**C**) Venn diagrams represent common gene presentation across transcriptome, proteome and ubiquitylome. (**D**–**F**) Pearson’s correlation (r) among (**D**) proteome & transcriptome (**E**) ubiquitylome and transcriptome (**F**) transcriptome and ubiquitylome. (**G**,**H**) L/H ratios for differentially expressed common genes from transcriptome, proteome, and ubiquitylome (**H**) along with heatmap representation (**G**).

The three kinds of omics data were integrated and analyzed to understand better the relationship among the transcriptome, proteome, and ubiquitylome of endocytosis-associated proteins and their correlation with HBV mediated pathogenesis. We also provided a summarized overview of the dynamics of the transcriptome, proteome, and ubiquitylome (Figure 4A). Based on the quant category, we further explored and compared the impact of HBV on the number of DEGs, DEPs, and Kub-sites involved in the endocytosis pathway (Figure 4B,C and Figure S1A). The number of DEGs and DEPs varied greatly, and only a few differentially expressed correlations were observed. The Pearson correlation coefficients were calculated as r = −0.2183 between proteome-transcriptome (Figure 4D), r = −0.019 between ubiquitylome–proteome (Figure 4E), and r = −0.013 for transcriptome and ubiquitylome (Figure 4F). We observed weak negative correlations in transcriptome-proteome, ubiquitylome–proteome and transcriptome–ubiquitylome. A similar result was observed by Spearman correlation coefficients (Supplementary Figure S1B–D). A summarized overview of correlation coefficients is described in Table 1. Furthermore, we analyzed and compared differentially expressed (*p* ≤ 0.05) 33 DEGs, DEPs, and Kub-sites which were common in all three-omics data sets (Figure 4G,H and Table 2). Notably, we noticed that prominent proteins such as HGS, EGFR, STX4, VAMP8, ICAM1, and SCAMP family members (SCAMP1 and SCAMP2) were found across the transcriptome proteome and ubiquitylome. Taken together, we observed weak negative associations in transcriptome-proteome, ubiquitylome–proteome and transcriptome–ubiquitylome.

### 2.5. Effects of HBV Infection on SCAMP1 mRNA, Protein, and Lysine Ubiquitination

**Figure 5:**
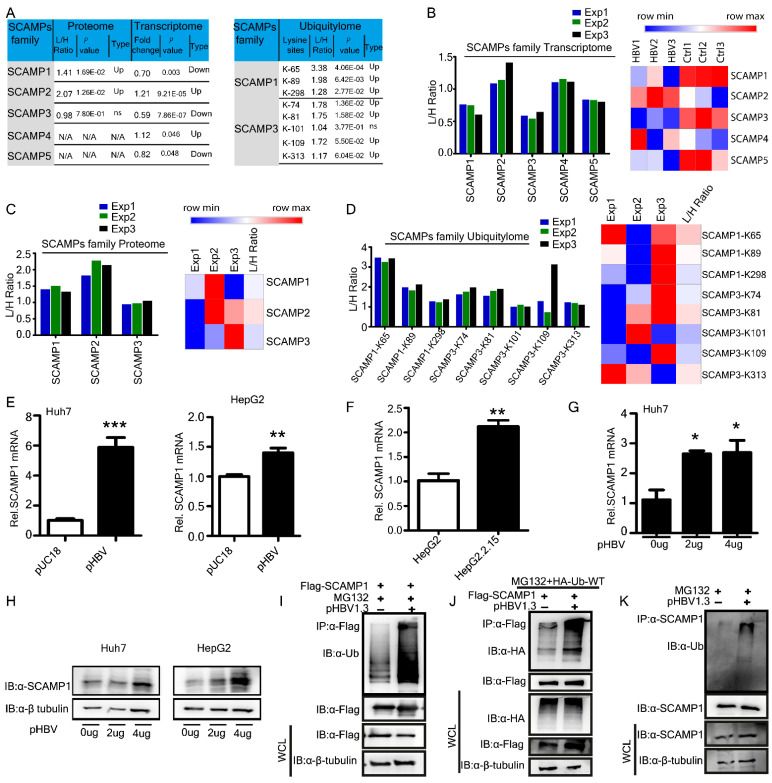
Host SCAMPs family transcriptome, proteome and ubiquitylome dynamics upon HBV integration. (**A**) Summary of quantified and differentially expressed SCAMPs family genes, proteins, and Kub-sites. (**B**–**D**) L/H ratios for SCAMPs family differentially expressed (**B**) transcriptome, (**C**) proteome, and (**D**) ubiquitylome from three biological replicates in HepG2.2.15 cells along with heatmaps representation, are shown. (**E**–**H**) Real-time PCR (**E**–**G**) and immunoblot analysis (**H**) of Huh7, HepG2, and HepG2.2.15cells, transfected with pUC18 empty vector or an increased dose of pHBV1.3, are shown. (**I**) Immunoprecipitation and immunoblot analysis of Huh7 cells transfected with FLAG-SCAMP1, Hepatitis B virus (HBV) 1.3-fold genome plasmid (pHBV1.3) and treated with MG132 are shown. (**J**) Co-immunoprecipitation and immunoblot analysis of extracts of Huh7 cells transfected with various combinations of plasmid for FLAG-tagged SCAMP1, HA-tagged wild type ubiquitin, Hepatitis B virus (HBV) 1.3-fold genome plasmid (pHBV1.3) and treated with MG132 is shown. (**K**) Immunoprecipitation and immunoblot analysis of Huh7 cells transfected with Hepatitis B virus (HBV) 1.3-fold genome plasmid (pHBV1.3) and treated with MG132 are shown, (0.01 < * *p* < 0.05; 0.001 < ** *p* < 0.01; and *** *p* < 0.001).

SCAMPs participate in endocytosis via a mechanism that may involve the recruitment of clathrin coats to the plasma membrane and the trans-Golgi network [22]. Here, we focused on SCAMP family members and summed up their modifications across omics data sets of the transcriptome, proteome, and ubiquitylome (Figure 5A). We observed that five members of the SCAMP family in the transcriptome were differentially expressed. SCAMP2 and SCAMP4 were significantly up-regulated, while SCAMP1, SCAMP3, and SCAMP5 were down-regulated (Figure 5B). In proteome, SCAMP1 and SCAMP2 were substantially augmented (Figure 5C). In the ubiquitylome analysis, SCAMP1-K65, SCAMP1-K89, SCAMP1-K298, SCAMP3-K74, SCAMP3-K81, SCAMP3-K109, and SCAMP3-K313 were found to be highly ubiquitinated (Figure 5D), as SCAMP1 was quantified and differentially expressed across all three omics data sets of the transcriptome, proteome, and ubiquitylome. So, we focused on investigating HBV-mediated effects on SCAMP1 mRNA and protein expression levels in Huh7, HepG2, and HepG2.2.15 cells. Surprisingly, HBV significantly enhanced SCAMP1 transcription, unlike our transcriptomic findings (Figure 5E,F). Further, we affirmed this up-regulation in a dose-dependent manner at mRNA and protein levels (Figure 5G,H). Next, we investigated HBV-induced changes in SCAMP1 ubiquitination through transfection to confirm HBV-induced changes in SCAMP1 ubiquitination. We co-transfected Flag-SCAMP1 expressing construct and HBV genomic DNA plasmid in Huh7 cells treated with MG132 and found that HBV markedly increased ubiquitination of SCAMP1 (Figure 5I). A similar ubiquitination pattern was observed in the presence of exogenous HA-tagged wild-type ubiquitin (Figure 5J). Next, we sought to determine ubiquitination under physiological conditions. Inconsistent with our observations, HBV significantly augmented endogenous ubiquitination of SCAMP1 (Figure 5K). These findings suggest that HBV up-regulates SCAMP1 RNA, protein, and ubiquitination.

### 2.6. Identification of SCAMP1 as a Novel Inhibitor of HBV Replication

**Figure 6:**
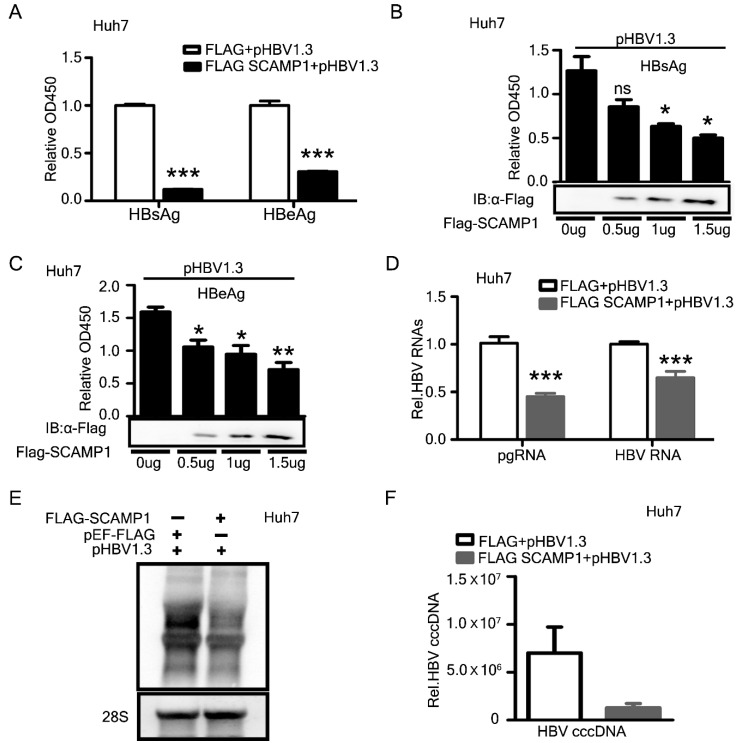
Overexpression of SCAMP1 inhibits HBV replication. Huh7 cells were transfected as indicated below and harvested 48 h after transfection (**A**–**F**). (**A**) ELISA determined the secreted HBsAg and HBeAg levels from the cell culture supernatants co-transfected (1:1) with pHBV1.3, FLAG-SCAMP1, and pEF-FLAG, are shown. (**B**–**D**) ELISA analysis of the secreted HBs Ag (**B**) and HBeAg (**C**) and Real-time PCR analysis of HBV RNAs or pgRNA (**D**) in Huh7 cells, transfected with pHBV1.3, together with pEF-FLAG or FLAG-SCAMP1, or an increased dose of FLAG-SCAMP1, are shown. (**E**,**F**) Northern blot analysis of HBV transcripts (**E**) qPCR analysis of HBV cccDNA (**F**) in Huh7 cells, transfected (1:1) with pHBV1.3, together with pEF-FLAG or FLAG-SCAMP1, are shown, (0.01 < * *p* < 0.05; 0.001 < ** *p* < 0.01; *** *p* < 0.001 and ns, not significant).

Among the endocytosis-associated proteins, SCAMP1 was quantified and differentially expressed across all three omics data sets of the transcriptome, proteome, and ubiquitylome. So we selected SCAMP1 to explore its previously unknown role in viral transcription and replication. We co-transfected the PEF-Flag-SCAMP1-expressing construct, along with pHBV1.3, into Huh7 and HepG2 cells, respectively, for analysis. ELISA analysis showed a markedly reduced amount of secreted HBsAg and HBeAg in supernatants from cultures of the transfected Huh7 or HepG2 cell lines (Figure 6A and Supplementary Figure S2A). Ectopic expression of SCAMP1 also inhibited secreted viral proteins in a dose-dependent manner (Figure 6B,C). Consistent with the reduction of viral secreted protein, the qPCR analysis showed a significant reduction in HBV pgRNA and viral mRNAs in both the transfected Huh7 or HepG2 cells (Figure 6D and Supplementary Figure S2B). Next, inhibition of HBV transcripts was confirmed by Northern blot analysis, indicating that SCAMP1 is most likely to inhibit HBV transcription (Figure 6E). Previously, over-expression of several host factors could inhibit HBV transcription and promote the decay of viral RNA [1,23,24]. Further, the qPCR analysis demonstrated that SCAMP1 suppressed HBV cccDNA replication in Huh-7 cells (Figure 6F). These results indicate that SCAMP1 over-expression decreases HBV production by inhibiting transcription and replication.

### 2.7. Knockdown of Endogenous SCAMP1 Enhances HBV Transcription and Replication

**Figure 7:**
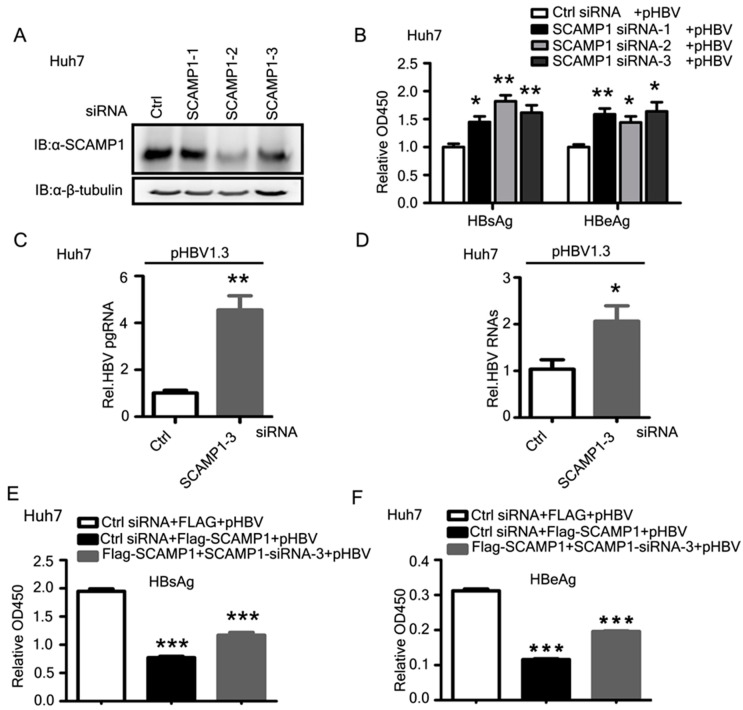
Knockdown of endogenous SCAMP1 leads to increased HBV replication. (**A**) Immunoblot analysis of SCAMP1 knockdown in Huh7 cells treated with SCAMP1-specific siRNAs or control (Ctrl) siRNA, β-tubulin serves as a loading control in all analyses. (**B**–**D**) ELISA analysis of secreted HBsAg and HBeAg (**B**) and Real-time PCR analysis of pgRNA (**C**) and HBV RNAs (**D**) in Huh7 cells, transfected with SCAMP1-specific siRNAs or control (Ctrl) siRNA or pHBV1.3, are shown. (**E**,**F**) The siRNA rescue experiment was performed in Huh7 cells, transfected with SCAMP1-specific siRNA or control (Ctrl) siRNA or pHBV1.3, together with pEF-FLAG or FLAG-SCAMP1, are shown. The complementation of SCAMP1 efficiently restored the amount of secreted HBsAg (**E**) and HBeAg (**F**), as determined by ELISA, (0.01 < * *p* < 0.05; 0.001 < ** *p* < 0.01; and *** *p* < 0.001).

To further determine the role of SCAMP1on HBV transcription and replication, we selected siSCAMP1 for efficiently knocking down the endogenous SCAMP1 expression in Huh7 cells or HepG2 cells (Figure 7A).

We co-transfected pHBV1.3 plasmid and siSCAMP1 or siRNA control into Huh7 cells. ELISA analysis showed that SCAMP1 knockdown significantly enhanced the amount of secreted HBsAg and HBeAg in the extracellular culture medium of cells (Figure 7B and Supplementary Figure S3A). Further, we showed that SCAMP1 knockdown markedly increased the amount of viral pgRNA and HBV RNAs (Figure 7C,D and Supplementary Figure S3B). To substantiate these findings and to rule out the possibility of any off-target effect from the SCAMP1 siRNA, we performed rescue experiments by co-transfecting PEF-Flag-SCAMP1 and SCAMP1 siRNA in Huh7 cells. As expected, the complementation of SCAMP1 efficiently restored the amount of secreted viral proteins in culture supernatants (Figure 7E,F). Collectively, these results suggest that SCAMP1 negatively regulates HBV replication and transcription.

### 2.8. SCAMP1 Downregulates HBV via Transcriptional Mechanisms

**Figure 8:**
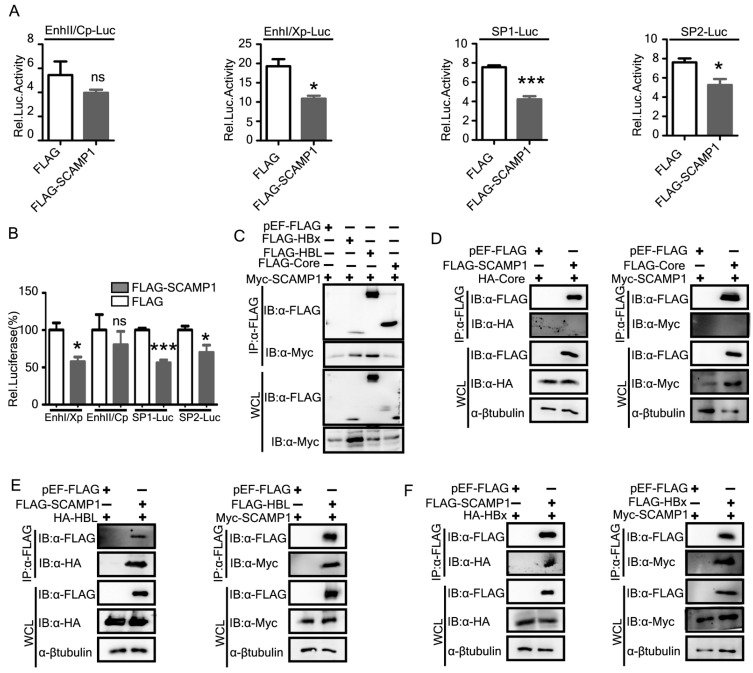
SCAMP1 reduces the HBV RNAs via transcriptional regulations. (**A**,**B**) HepG2 cells were co-transfected with four reporter plasmids individually and SCAMP1 expression plasmid or its control vector. The cells were lysed 48 h post-transfection and subjected to luciferase activities assay. For each transfection, pRL-TK was included as a control for transfection efficiencies. The activity of EnhI/Xp, SP1, and SP2 was suppressed by SCAMP1. (**C**–**F**) Co-immunoprecipitation and immunoblot analysis of Huh7 cells transfected with constructs expressing Myc-SCAMP1, HA-tagged HBV Core, X, L, or FLAG-tagged HBV Core, X, L, or pEF-FLAG, respectively are shown, (0.01 < * *p* < 0.05; *** *p* < 0.001 and ns, not significant).

The pgRNA of HBV serves as the template for reverse transcription and is critical for viral replication [25]. HBV transcription is controlled by four promoters (preS 1, preS 2, Core, and X) and two enhancers (enhancer Ι and enhancer II), which are entirely dependent on host transcriptional factors [26]. SRY-Box Transcription Factor 9 (SOX9) mediated repression of HBV replication by binding to and inhibiting HBV EnhII/Cp [27]. Hepatocyte nuclear factor6 (HNF6) suppresses the activity of the preS2/S promoter in a dose-dependent manner [1]. The HBV enhancer and promoter (EnhI/Xp, EnhII/Cp, preS1p, and preS2p, respectively) activities in Sirt2 knockdown cells were lower than those in mock- and controlled shRNA-transduced Huh7 cells [28]. Since SCAMP1 inhibited both pgRNA and HBV RNAs, therefore we sought to determine the effects of SCAMP1 on four HBV promoters by a luciferase-based reporter assay. Huh7 cells were co-transfected with SCAMP1 expressing plasmid along with the reporters driven by EnhI/Xp, EnhII/Cp, SP1, and SP2 promoters. We found that SCAMP1 overexpression significantly inhibited the activity of the HBV EnhI/Xp, SP1, and SP2 promoters and showed a modest effect on EnhII/Cp (Figure 8A,B). The modest effects on EnhII/Cp might be attributed to the differential regulation of precore RNA and pgRNA, as described previously [23,29]. These results indicate that SCAMP1 suppressed the activity of the EnhI/Xp, SP1, and SP2 promoters transcriptionally, which accounts for the inhibition of X and S mRNA, respectively. It is reported that SP1 can serve both as a positive and negative regulator for the expression of HBV genes [30]. To further investigate the mechanisms of the inhibitory effects of SCAMP1 on the HBV, we performed co-immunoprecipitation and immunoblot analysis in Huh7 cells expressing SCAMP1, HBV X, L, or Core proteins. We found that SCAMP1 interacted with L and X but not with Corein reciprocal co-IP experiments (Figure 8C–F). The TRIM14 SPRY domain interacts with the C-terminal of HBx and inhibits the HBV replication [31]. Hepatocystin functions as a chaperon-like molecule by accelerating HBx degradation via binding its C-terminal [32]. Our findings revealed that SCAMP1 was physically associated with viral and X and L and might be involved in HBV inhibition via suppression of these proteins. Taken together, the effect of SCAMP1 on viral transcription is by the specific transcriptional inhibition of HBV EnhI/Xp, SP1, and SP2 promoters, rather than from a general or global effect on human hepatoma cells.

## 3. Discussion

Endocytosis is one of the most studied conserved processes which maintain and regulate receptor-mediated signaling pathways initiated from plasma membrane receptors. Viruses take advantage of the endocytosis machinery to reach intracellular compartments and exploit the network of endocytic organelles for penetration into the cytosol or replication sites. HBV internalization into cells is triggered by virus–receptor interactions in an endocytosis-dependent manner (mainly clathrin-mediated endocytosis) [5,6]. A significant number of studies are available regarding the HBV-mediated alterations in the transcriptome [33], the proteome of host cell lipid rafts [34], combined proteomics and metabolomics analyses [35]. Recently, using SILAC-based mass spectrometry (MS) analysis, we reported host global ubiquitylome and proteome modifications induced by HBV infection [17]. However, HBV-mediated changes in the host endocytosis pathway were not analyzed in the transcriptome, proteome, and ubiquitylome to better understand the complex interactions of HBV and host factors. In this study, we conducted RNA-sequencing, quantitative mass spectrometry, and Ubiscan analysis and thoroughly explored the DEGs, DEPs, and Kub-sites involved in the endocytosis pathway.

In the transcriptome, we found 273 quantified genes; 36 genes were changed over two folds which were 13.1% of quantified genes. The most significant one among the two-fold up-regulated genes was RAB31, with a fold change of ≈15.66. HBV-mediated differential expression of RAB31 was reported in another study [36]. Similarly, across the 2-folds down-regulated genes, the most considerable one was LRP2, with the lowest fold change of ≈0.01. Notably, HBx down-regulates tumor suppressor DAB2 via increased expression of miR-106b [37].

In proteome, 18% of endocytosis-associated host proteins were changed over two-folds, which was comparatively higher than the 13.1% found in the transcriptome. According to subcellular localization analysis, most of these proteins were located in the cytoplasm, suggesting that HBV infection primarily modifies those endocytic proteins localized in the cytoplasm. Viral infection causes changes in gene expression and the subcellular localization of some host proteins. These changes may support or inhibit virus accumulation and spreading. Influenza A virus (IAV) changes host cell protein abundances and subcellular localization [38]. ICAM-1 is an adhesion molecule highly expressed in chronic hepatitis B infection and found to be associated with hepatocellular carcinoma [19,20]. CHMP6 is a component of the endosomal sorting complex required for transport (ESCRT III) which plays a crucial role in the budding of HBV [39]. Several studies reported that overexpression of full-length IGF2R decreases cell growth and decreases tumor growth in vivo [40,41,42]. However, overexpression of IGF2R has also been associated with an increase in cell number [43]. It has also been reported that hepatic sortilin (SORT1) is inversely associated with HBsAg expression in hepatocytes [44]. Increased risk of hepatocellular carcinoma was found in NPC patients, suggesting a need for screening in this patient population [45]. We observed a distinct down-regulation of IQGAP1and LGALS3 in our SILAC analysis, but astonishingly, In contrast to our findings, IQGAP1and LGALS3was markedly up-regulated in HBV-positive patients and positively correlated to poor prognosis of HBV-associated HCC patients [46,47,48].

In ubiquitylome, we identified 221 ubiquitinated Lys sites in 77 endocytosis-associated host proteins with modified ubiquitination in response to HBV infection. In this study, we, for the first time, demonstrated the ubiquitination change of endocytosis-associated secretory carrier-associated membrane protein 1 (SCAMP1) and also revealed its novel role in HBV transcription and replication. We found SCAMP1 to be highly ubiquitinated at positions K65 and K89 over two-fold. Subsequently, we confirmed the altered ubiquitination of the endocytosis-associated protein SCAMP1. Previously, HBV increased the ubiquitination of VAMP8 and HGS whereas decreasing the ubiquitination of VAMP3, HSPA8 and RAB8A, which are essential proteins associated with the endocytosis pathway that contribute to membrane transport [17]. We screened 10 hub genes in endocytosis-associated proteome through PPI analysis and found that transferrin receptor protein 1 was the most outstanding hub gene in proteome while in the ubiquitylome most significant hub protein was the epidermal growth factor receptor (EGFR). Transferrin receptor 1 (TFRC 1), which mediates the virus entry, its overexpression is observed in hepatocellular carcinoma (HCC) [49].

Viral infection causes changes in host gene expression and the subcellular localization of some host proteins. These changes may support or inhibit virus accumulation and spreading. According to the sub-cellular localization analysis, most proteins were localized in the cytoplasm and contributed approximately 51% and 41% in proteome and ubiquitylome, respectively. It is reported that the most frequent change is the movement of host cytoplasmic proteins into the sites of virus replication via interactions with viral proteins [50].

We further correlated and compared the impact of HBV on 33 common DEGs, DEPs, and Kub-sites which were involved in the endocytosis pathway. In response to HBV infection, we observed weak negative correlations in transcriptome-proteome, ubiquitylom-proteome and transcriptome-ubiquitylome. Spearman correlation coefficients observed a similar result. The discordance of transcriptome and proteome might be due to post-transcriptional regulations likely to play a significant role in determining protein accumulation. In a previous study, genes involved in metabolic pathways had the strongest positive mRNA-protein correlations, while those involved in cell-cycle and mRNA pathways had weaker correlations, indicating major post-transcriptional regulations. The conflict between mRNA and protein abundance was identified in 16 genes which were components of respiratory chain complex 1 in response to HBV infection [51]. It implies that concordance and discordance between transcriptome and proteome depend on post-transcriptional regulations and vary from a pathway to pathway.

Since the discovery of SCAMPs, their functions have been studied extensively in mammalian cells in subcellular localization and membrane trafficking for more than two decades [10,22]. SCAMPs participate in endocytosis via a mechanism that may involve the recruitment of clathrin coats to the plasma membrane and the trans-Golgi network [22]. In this study, we explored the changes in SCAMP family members upon HBV infection across the transcriptome, proteome, and ubiquitylome. Hence, we investigated HBV mediated effects on SCAMP1 mRNA and protein expression levels in Huh7, HepG2, and HepG2.2.15 cells. Astonishingly, HBV significantly enhanced SCAMP1 transcription, unlike our transcriptomic findings, but the up-regulation of SCAMP1 protein corresponded with our SILAC findings. In our previous study, few proteins showed conflict with omics analysis, suggesting that quantitative omics analysis conveyed relatively reliable information regarding the global proteome and ubiquitylome of cells. However, it is not a complete and absolute scanning method [17]. We confirmed the ubiquitination of SCAMP1 mediated by HBV infection for the first time in this study. In addition to its membrane trafficking and cancer-related function, we demonstrated the previously unknown antiviral function of SCAMP1 against HBV infection. The overexpression of SCAMP1 reduced, whereas the suppression of endogenous SCAMP1 increased HBV transcripts and viral proteins (Figure 6 and Figure 7). Previous studies showed that EGF modulated HBV infection dose-dependently via EGFR-mediated endocytosis pathways [52,53]. HBsAg expression was found to be more than 10-folds suppressed upon SCAMP1 over-expression (Figure 6A and Supplementary Figure S2A). HBV envelope protein S is essential for viral particle formation, infectivity, and immune response [54]. Another endocytosis-associated protein, VAMP8 overexpression, reduced the levels of secreted HBsAg and intracellular HBV DNA [55]. In rescue experiments of SCAMP1 knockdown, complementation of SCAMP1 expression efficiently reduced the viral production, implying SCAMP1 as an essential negative regulator of HBV infection. Aberrant levels of endocytosis-associated protein HGS suppressed HBV transcription, replication and virion secretion [56]. The cccDNA serves as the transcription template of pregenomic RNA (3.5 kb) and other mRNAs, including precore RNA (3.5 kb), S RNAs (2.4/2.1 kb), and X RNA (0.7 kb) [57]. pgRNA acts as the template for translation of the viral polymerase and core protein and the template RNA, which is reverse transcribed into viral DNA [58]. As in our results, SCAMP1 inhibited HBV cccDNA, pgRNA, and viral mRNAs (Figure 6E,F and Supplementary Figure S2B). Thus, we hypothesized that SCAMP1 might have inhibited HBV transcriptionally. So, by performing reporter assays, we observed that SCAMP1 suppressed the activity of the EnhI/XP, SP1, and SP2 promoters, which evidenced HBV inhibition at the transcriptional level. Previously, host factors such as hepatocystin and TRIM14 destabilized the HBx protein via binding the C-terminal of HBx [31,32]. It is reported that HNF6 suppressed HBsAg expression by both transcriptional and post-transcriptional mechanisms via inhibiting the SP2 promoter [1]. Similarly, ZAP mediated HBV RNA reduction post-transcriptional [24].

Altogether, we have presented a multi-omics landscape of the HBV-host interaction through SILAC, RNA-sequencing, and Ubiscan analysis. We demonstrated HBV-induced changes in the endocytosis-associated transcriptome, proteome, and ubiquitylome. The majority of HBV-modified host proteins were subcellularly localized in the cytoplasm. A weak negative correlation was observed among transcriptome, proteome and ubiquitylome. Remarkably, SCAMPs family members, particularly SCAMP1, were differentially expressed in all three omics-datasets. Our results demonstrated a novel function of SCAMP1 against HBV replication and gene expression. These findings have opened new opportunities to identify potential drug targets or antiviral agents, showcased with SCAMP1. More omics work should be performed to develop novel therapeutics and potential diagnostics in combating HBV infection to better understand host–virus interaction.

## 4. Materials and Methods

### 4.1. Cell Lines and Transfection

HepG2and Huh7 cells were kept in our lab. All cells were cultured in DMEM (Gibco/Life Technologies, Thermo Fisher Scientific, Waltham, MA, USA), containing 10% FBS (Gibco/Life Technologies). According to the manufacturer’s protocol, cell transfection was done using Lipofectamine 2000 (Invitrogen, Thermo Fisher Scientific, Waltham, MA, USA).

### 4.2. RNA Extraction, Library Preparation, and Sequencing

According to the manufacturer’s instructions, the total RNA was extracted by Trizol (Invitrogen). An amount of 2 μg of total RNAs was used for stranded RNA sequencing library preparation using Stranded mRNA Library Prep Kit for Illumina^®^ in three distinct biological replicates that proceeded independently (Wuhan Seqhealth Technology Co., Ltd., Wuhan, China) following the manufacturer’s instructions. PCR products corresponding to 200–500 bps were enriched, quantified, and finally sequenced on the Hiseq6000 sequencer (Illumina). They were mapped to the reference genome of Homo sapiens using STRA software (version 2.5.3a) with default parameters. Reads mapped to the exon regions of each gene were counted by feature Counts (Subread-1.5.1; Bioconductor), and then RPKMs were calculated. Genes differentially expressed between groups were identified using the edge R package (version 3.12.1). An FDR corrected *p*-value cutoff of 0.05, and a Fold-change cutoff of 2 was used to judge the statistical significance of gene expression differences. Gene ontology (GO) analysis and Kyoto encyclopedia of genes and genomes (KEGG) enrichment analysis for differentially expressed genes were both implemented by KOBAS software (version: 2.1.1) with a corrected *p*-value cutoff of 0.05 to judge statistically significant enrichment. Alternative splicing events were detected using rMATS (version 3.2.5) with an FDR value cutoff of 0.05.

### 4.3. SILAC Labelling, Protein Extraction, and Trypsin Digestion

We performed SILAC labelling, protein extraction, and trypsin digestion according to previously described protocols [17]. The HepG2-K6R10 cells were labelled with the “heavy” form of L-^13^C_6_-lysine/L-^13^C_6_^15^N_4_-arginine. In contrast, the HepG2.2.15-K0R0 cells were labelled with the “light” form L-^12^C_6_-lysine/L-^12^C_6_^14^N_4_-arginine separately using a SILAC Protein Quantitation Kit in three distinct biological replicates that proceeded independently (Jingjie PTM BioLab, Co., Ltd., Hangzhou, China) according to manufacturer’s instructions. Briefly, the cells were grown in DMEM medium supplemented with 10% fetal bovine serum for more than seven generations before being harvested to achieve more than 97% labelling efficiency.

The harvested “heavy” and “light” labelled cells were sonicated three times on ice using a high-intensity ultrasonic processor (Scientz, Ningbo, China) in lysis buffer (8 M Urea, 5 mM DTT, 2 mM EDTA, 1.0% cocktail III, and 50 μM PR619). The remaining debris was removed by centrifugation at 20,000× *g* at 4 °C for 10 min. After concentration measurement, equal amounts of crude proteins in the supernatant were labelled. “Heavy” or “light” was mixed, and the crude proteins were precipitated with TFA using a 15% final concentration (*v*/*v*) (soluble fraction). After washing twice with −20 °C acetone, the protein pellets were dissolved in 100 mM NH_4_HCO_3_ (pH 8.0) for trypsin digestion. Trypsin solution (Promega) (trypsin: protein = 1:50) was added to proteins, and then the protein pellets were digested at 37 °C for 16 h. After the alkylation reaction, trypsin (trypsin: protein = 1:100) was added again and incubated (37 °C, 4 h).

#### 4.3.1. HPLC Fractionation and Affinity Enrichment

Tryptic hydrolysis was followed by separating the sample into 80 fractions through high pH reverse-phase HPLC using Agilent, USA 300 Extend C18 column (5 µm particles, 4.6 mm ID, 250 mm length). The tryptic peptides were dissolved in NETN mixture (0.5% NP-40, 1 mM EDTA, 100 mM NaCl, 50 mM Tris-HCl, pH 8.0) and incubated with pre-washed antibody beads (PTM Biolabs, Hangzhou, China) overnight at 4 °C with gentle shaking. Peptides-enriched beads were washed 4 times with NETN and twice with ddH_2_O. The combined peptides were eluted from the beads with 0.1% TFA, collected, vacuum dried, and cleaned using C18 ZipTips (Millipore, Germany).

#### 4.3.2. LC-MS/MS Analysis and Database Search

Peptides were dissolved in 0.1% FA and directly loaded onto a reversed-phase pre-column (Acclaim PepMap 100, Thermo Scientific, Waltham, MA, USA). A reversed-phase analytical column (Acclaim PepMap RSLC, Thermo Scientific, Waltham, MA, USA) was used to separate peptide fractions, with a gradient of an increase in solvent B (0.1% FA in 98% ACN) from 6% to 22% for 26 min, 22% to 35% for 8 min, went up to 80% in 3 min and staying at 80% for another 3 min. The whole process happened at a constant flow rate of 300 nL/min on an EASY-nLC 1000 UPLC system. The following analysis of resulting peptides was carried out by Q Exactive^TM^ Plus hybrid quadrupole-Orbitrap mass spectrometer (ThermoFisher Scientific, Waltham, MA, USA). According to the previously described protocol, we performed MS/MS data processing using MaxQuant with an integrated Andromeda search engine (v. 1.5.2.8) [17]. Tandem mass spectra were searched against the Swiss-Prot human database concatenated with reverse decoy database. Trypsin/P was specified as a cleavage enzyme for proteomic peptides, allowing up to 2 missing cleavages, 5 modifications per peptide, and 5 charges. Carbamidomethylation on Cys was specified as fixed modification, oxidation on Met, and acetylation on protein N-terminal were specified as variable modifications. All the other parameters in MaxQuant were set to default values.

For peptides with ubiquitinated Lys sites, Trypsin/P was specified as cleavage enzyme allowing up to 4 missing cleavages, 5 modifications per peptide, and 5 charges. Carbamidomethylation on Cys was specified as fixed modification and oxidation on Met, GlyGly on Lysine, and oxidation on Met was specified as variable modifications. All the other parameters in MaxQuant were set to default values. Te site localization probability was set as ≥ 0.75.GO annotation was analyzed through the UniProt-GOA database and InterProScan software. WoLF PSORT was applied to predict subcellular localization. Volcano plots Venn diagrams were drawn inTBtools, and heatmaps were drawn in MORPHEUS.

### 4.4. si-RNAs and Plasmid Construction

SCAMP1-siRNAs and negative control siRNAs (si-NC) were synthesized by RiboBio (Guangzhou, China). In order to generate the FLAG-SCAMP1, FLAG-SCAMP1-K65R, and Myc-SCAMP1 constructs, the corresponding cDNAs were cloned into the pEF-FLAG or pEF-Myc. Constructs like FLAG-HBx, FLAG-HBs, FLAG-HBc, HA-HBx, HA-HBs, HA-HBc pXJ40-HA, pUC18, and pHBV1.3 were already available in our laboratory. All siRNA and generated constructs primer sequences are listed in Table S1.

### 4.5. RNA Extraction and qRT-PCR

Total RNA was extracted using TRIzol reagent (TaKaRa, Shiga, Japan), and reverse transcription was carried out using the Prime Script RT Reagent Kit with gRNA Eraser (TaKaRa, Shiga, Japan), according to the manufacturer’s instructions. All reactions were carried out in duplicate. GAPDH was set as an internal control for protein-coding gene expression in each cell line and sample. qRT-PCR analyses were carried out as previously described [59,60].

### 4.6. In Vivo Ubiquitination Assay

We performed immunoprecipitation according to previously described protocols [61]. Cells were cultured in a 60-mm dish and treated with 10μM MG132 for 2–4 h. Cells were lysed after 48 h with Lysis Buffer (50 mMTris-Cl [pH 8.0], 150 mM NaCl, 0.1% SDS, 1% NP-40, 0.5% Sodium deoxycholate) supplemented with 1% cocktail and freshly dissolved 10 mM N-ethylmaleimide (NEM). After centrifugation, the supernatant was immunoprecipitated with Flag-antibodies and protein A/G beads. Then the mixture was incubated overnight at 4 °C. The beads were washed 3 times with washing buffer (20 mMTris-Cl [pH 7.5], 150 mM NaCl, 0.5% NP-40 mM ethylene diamine tetraacetic acid). The proteins bound to the beads were analyzed by western blot with anti-Ub- antibodies.

### 4.7. Immunoprecipitation and Immunoblot Analyses

Immunoprecipitation and immunoblot analyses were performed as previously described [59]. Briefly, proteins were separated by SDS-PAGE, transferred to nitrocellulose membranes, and then blocked with 5% skim milk. Blots were then incubated with specific primary antibodies overnight at 4 °C; these included antibodies to detect FLAG, β-tubulin, HA, Myc and GAPDH (Sigma, St Louis, MO, USA) and SCAMP1 (Proteintech, Wuhan, China), then HRP-conjugated secondary antibody (Sigma, St Louis, MO, USA) at room temperature for 1 h. Immunoreactivity of protein was visualized through ECL western blotting kit (Millipore, Hercules, CA, USA). Cell suspensions were mixed with either FLAG beads or a mixture of HA antibody and Protein A/G agarose for co-IP assays. After incubation at 4 °C overnight, the FLAG or Protein A/G beads were washed 3 times with washing buffer (50 mM Tris HCl, 150 mM NaCl, pH7.5), and the immune complexes were eluted from the beads. SDS-PAGE and western blot analysis was then used to separate and identify the eluted proteins.

### 4.8. Enzyme-Linked Immune-Sorbent Assay

The hepatitis B surface antigen (HBsAg, diluted 1:50) and hepatitis B e antigen (HBeAg, diluted 1:30) viral-secreted proteins in the culture supernatant were determined using enzyme-linked immunosorbent assay (ELISA) kits (Kehua Bio-engineering, Shanghai, China).

### 4.9. Northern Blot Analysis

The extracted cellular RNA was detected using the DIG Northern starter kit (Roche Diagnostics, Indianapolis, IN) for northern blot corresponding to nucleotides 1072 to 2171 of the HBV genome [60].

### 4.10. Reporter Assay

The reporter plasmids EnhI/X (nt 950-1375), EnhII/C (nt 1415-1815), SP1 (2707-2849), SP2 (2937-3182) were inserted into the SacI, and HindIII restriction sites of pGL3-Basic (Promega, Madison, WI, USA) were already available in our laboratory. The reporter assay was performed with co-transfection of 0.1 μg Flag-SCAMP1, 0.1 μg pEF-FLAG 0.1μg pGL3-XpLUC, 0.1μg pGL3-CpLUC, 0.1μg pGL3-PS1pLUC, 0.1μg pGL3-SpLUC into HepG2 cells in 24-well plates in triplicates in designated experimental settings. After 48hours post-transfection, the firefly luciferase expression driven by the HBV promoter was first normalized to the renilla luciferase expression driven by the TK promoter. The cells were lysed with 450 uL lysis buffer and subjected to luciferase activity assays using the Dual-Glo system (Promega, WI, USA).

### 4.11. Protein-Protein Interaction (PPI) Network Analysis Construction

The Search Tool for the Retrieval of Interacting Genes (STRING) database is a global resource of protein-protein interactions with a confidence score to quantify each interaction confidence. (http://string-db.org, accessed date 28 August 2021). Molecular interaction networks were visualized with Cytoscape (version 3.8.2), an open-source bioinformatics software platform. The species for PPI analysis was set as a human with the interaction score ≥0.4. Nodes in the PPI network represented the genes, and edges between nodes represented the interactions between the genes.

### 4.12. Statistical Analysis

All statistical analyses were carried out using GraphPad Prism 6 software. Data are presented as mean ± (SEM), and a two-tailed Student’s *t*-test (two-sample equal variance) was used to measure the significance of observed differences between two groups. The data were expressed as mean ± (SEM) and *p* < 0.05 was considered to be statistically significant (0.01 < * *p* < 0.05; 0.001 < ** *p* < 0.01; and *** *p* < 0.001) 

## Figures and Tables

**Table 1:** Correlation coefficients for comparison of changes in protein, ubiquitin, and RNA levels in response to HBV infection.

Parameters	Proteome−Transcriptome	Ubiquitylome−Proteome	Transcriptome−Ubiquitylome
Pearson r	−0.2183	−0.01983	−0.01331
*p* value	0.003	0.7634	0.8505
Spearman r	−0.1456	−0.03623	−0.00135
*p* value	0.0492	0.5822	0.9846
R squared	0.04765	0.00039	0.00017
Number of XY pairs	183	233	203

**Table 2:** Endocytosis associated differentially expressed common genes in the Transcriptome, Proteome, and Ubiquitylome in response to HBV infection.

Common Genes		Transcriptome		Proteome		Ubiquitylome		
Protein Accession	Gene Name	FCs	Regulation	L/H Ratio	Regulation	Lysine Position	L/H Ratio	Regulation
O15126	SCAMP1	0.74	Down	1.41	Up	65	3.38	Up
O14828	SCAMP3	0.63	Down	0.98	Down	74	1.78	Up
O94973	AP2A2	0.6	Down	1.15	Up	6	1.29	Up
P61421	ATP6V0D1	1.42	Up	1.69	Up	343	0.63	Down
P27824	CANX	0.6	Down	2.56	Up	458	1.64	Up
Q7LBR1	CHMP1B	1.72	Up	0.61	Down	104	0.54	Down
P50570	DNM2	0.7	Down	1.51	Up	223	0.46	Down
P00533	EGFR	0.21	Down	0.68	Down	708	2.34	Up
Q9Y6I3	EPN1	0.58	Down	1.47	Up	128	0.45	Down
Q9UBC2	EPS15L1	0.67	Down	1.24	Down	838	3.34	Up
O14964	HGS	0.7	Down	1.79	Up	226	4.45	Up
P11142	HSPA8	0.41	Down	0.84	Down	539	2.26	Up
P05362	ICAM1	1.86	Up	28.4	Up	524	0.26	Down
P46940	IQGAP1	1.45	Up	0.16	Down	1083	0.41	Down
Q96J02	ITCH	1.14	Up	0.83	Down	350	3.23	Up
Q9NZM3	ITSN2	1.59	Up	1.68	Up	583	1.93	Up
P19338	NCL	1.98	Up	0.86	Down	324	0.2	Down
Q96PU5	NEDD4L	1.27	Up	1.49	Up	462	2.33	Up
P15531	NME1	0.65	Down	0.72	Down	12	0.79	Down
O15118	NPC1	1.24	Up	4.71	Up	1180	1.54	Up
P49757	NUMB	2.01	Up	0.95	Down	174	5.7	Up
Q13393	PLD1	3.8	Up	1.21	Up	796	1.96	Up
P61026	RAB10	2.23	Up	1.14	Up	102	0.27	Down
P61006	RAB8A	0.64	Down	1.31	Up	138	0.29	Down
Q14160	SCRIB	0.72	Down	0.39	Down	53	0.47	Down
P23246	SFPQ	0.62	Down	1.36	Up	413	1.41	Up
Q99961	SH3GL1	0.76	Down	1.4	Up	20	1.13	Up
O60493	SNX3	1.21	Up	1.25	Up	119	0.77	Down
O75886	STAM2	1.41	Up	0.91	Down	202	2	Up
Q12846	STX4	1.49	Up	1.7	Up	243	0.42	Down
P02786	TFRC	1.26	Up	0.22	Down	53	2.06	Up
Q9BV40	VAMP8	3.63	Up	0.8	Down	47	2.46	Up
Q96QK1	VPS3	0.74	Down	0.87	Down	694	0.84	Down

## Data Availability

The datasets used and analyzed during the current study are available in detail from the corresponding author on reasonable request.

## Supplementary Materials

The following supporting information can be downloaded at: https://www.mdpi.com/article/10.3390/ijms23042211/s1.
