# Supplementary figures and images for: Multiomics Analysis of Endocytosis upon HBV Infection and Identification of SCAMP1 as a Novel Host Restriction Factor against HBV Replication
Source: Int J Mol Sci. 2022 Feb 17;23(4):2211. doi: 10.3390/ijms23042211 (PMC8874515; doi:10.3390/ijms23042211)

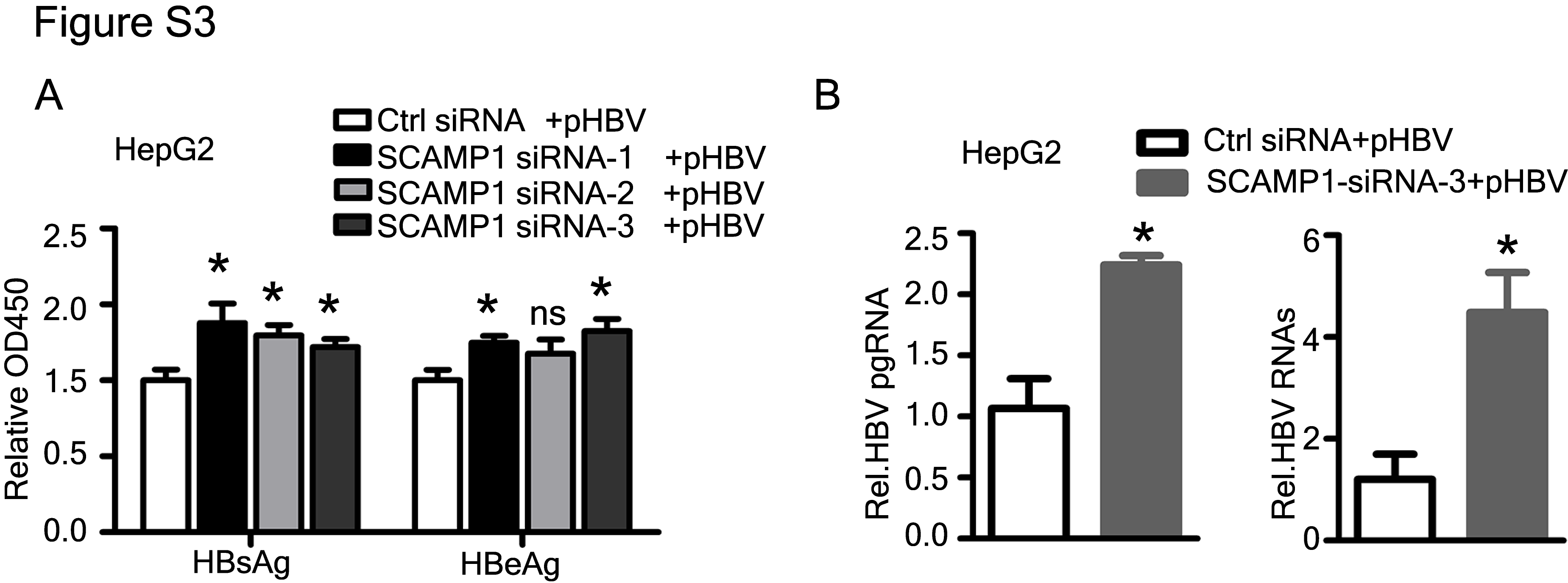

Supplement: Supplementary file 1 [file ijms-23-02211-s001.zip › Supplementary Figure S3.tif]

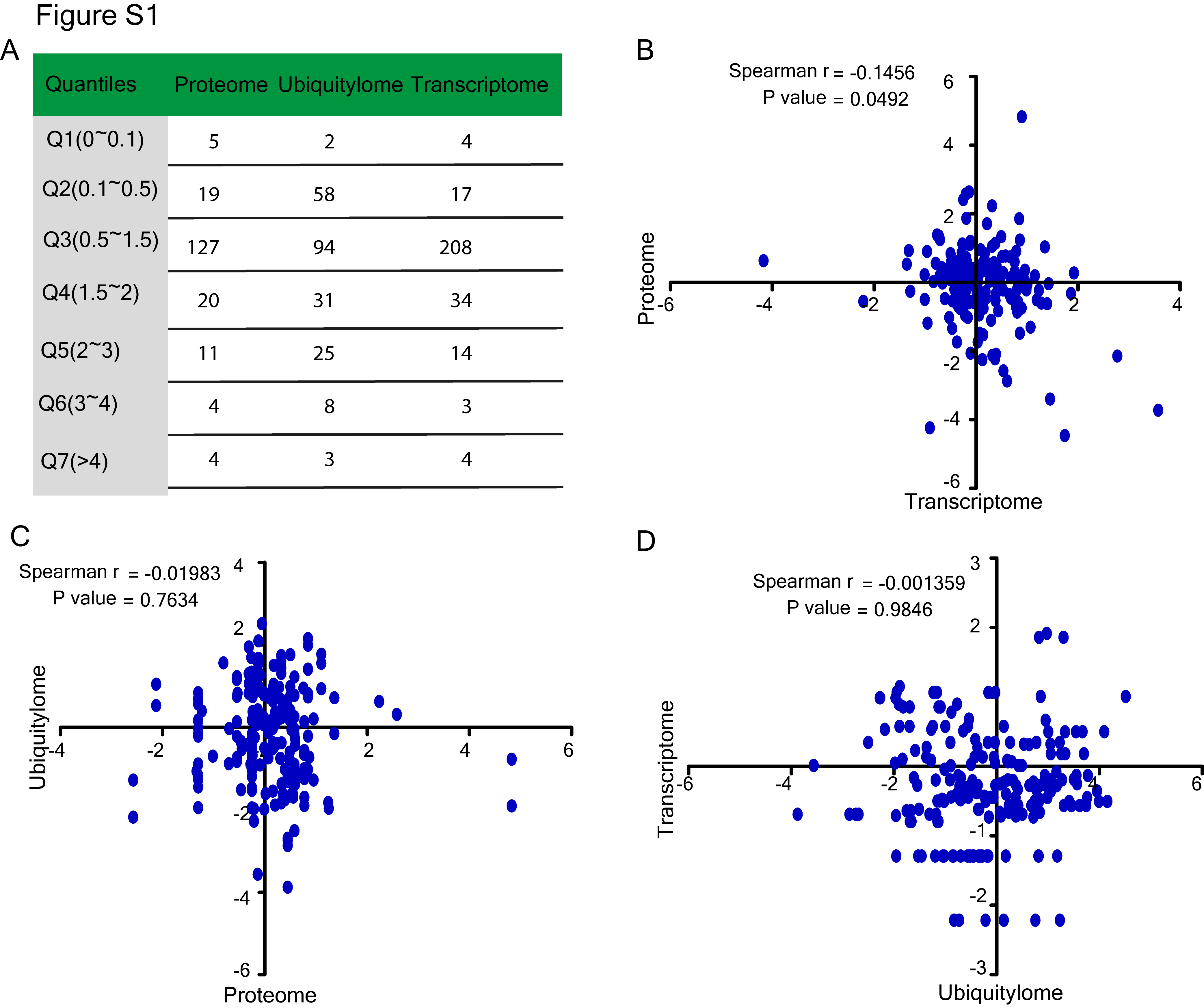

Supplement: Supplementary file 1 [file ijms-23-02211-s001.zip › Supplementary Figure S1.tif]

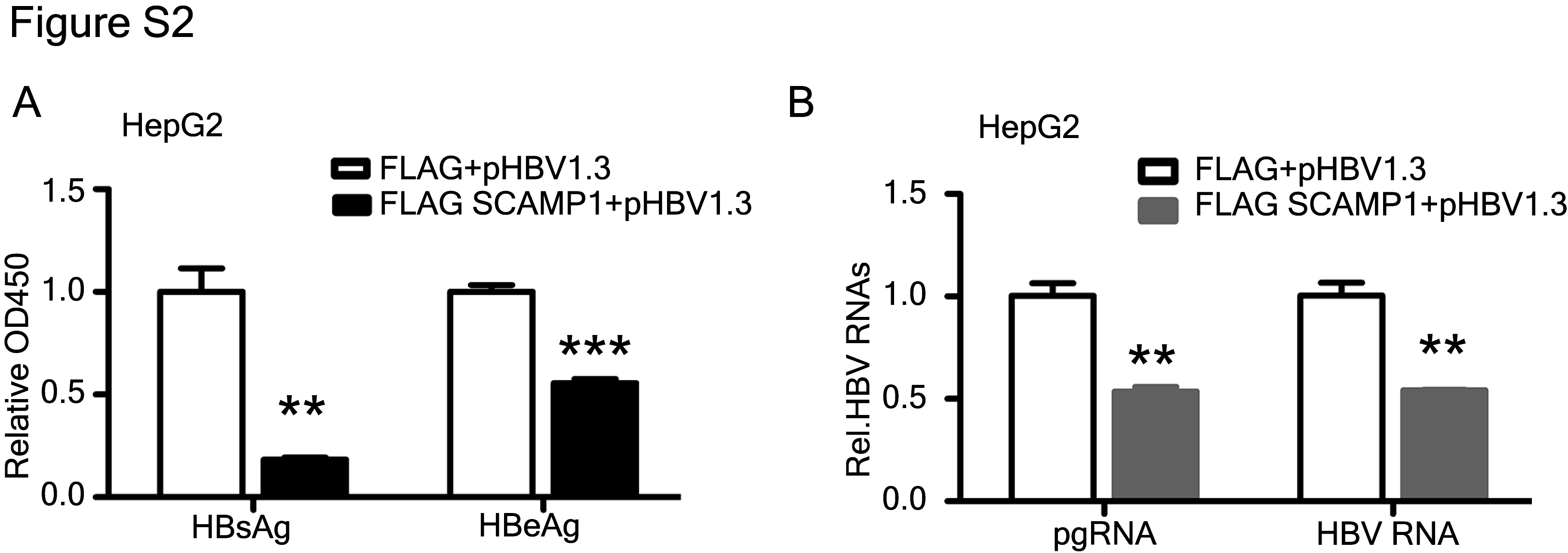

Supplement: Supplementary file 1 [file ijms-23-02211-s001.zip › Supplementary Figure S2.tif]
